# Children in relation to agricultural health and safety in Sweden: A perspective

**DOI:** 10.3389/fpubh.2022.1070027

**Published:** 2023-01-11

**Authors:** Peter Lundqvist

**Affiliations:** Department of People and Society, Swedish University of Agricultural Sciences, SLU Alnarp, Lomma, Sweden

**Keywords:** children, adolescents, parents, rural, agriculture, farm, Sweden

## 1. Background

Rights of children have been an important issue in Sweden for many years. The Ombudsman for Children is a government agency tasked with promoting and advancing rights and interests of children in Sweden on the basis of the UN Convention on the Rights of the Child (1). In 1979, Sweden was the first country in the world that made it illegal to hit children, both at home and in school (2). Sweden is also well-known for its efforts to protect children in traffic, with measures such as the rear-facing car seat (3).

Sweden is a country with <60,000 farms, dominated by family operated farms. Their children grow up in an environment that includes not only a significant experience of living close to nature, but also being close to a workplace that might be both risky and unhealthy. In the old days, the farms consisted of many people who were always available to share the care of children. However, nowadays, often one parent works away from home and small children go to daycare or stay with one parent on the farm. There are no official statistics on child injuries on farms, but there is reporting by the media on time to time serious injuries and fatalities among children as well as adolescents by farm tractors and machinery, ATVs, horses, and other large animals (4).

Children on farms and in rural areas have been an important component of different health and safety programs, but research in this area has been limited. Inspiration for Swedish initiatives and actions often came from US organizations such as the National Children's Center for Rural and Agricultural Health and Safety in Marshfield, WI, Farm Safety 4 Just Kids, and the Progressive Agriculture Safety Days, as well as participation in the Childhood Agricultural Safety Network. In 1996, Swedish activities took on this issue of sharing challenges and possible solutions regarding children on farms during the US–Nordic Conference on Rural Childhood Injury Prevention in Stockholm (5).

## 2. Current situation and activities

There are no current national programs or dedicated organizations focused on childhood agricultural safety in Sweden. Despite this fact, there have been relevant activities, both among farming organizations as well as among researchers. The most relevant activities are presented briefly:

### 2.1. Children and health and safety campaigns

When there was an occupational health service (OHS) for farmers in Sweden (Lantbrukshälsan) they held a “Year of Rural Child Safety” in 1996, which included a variety of activities. After the OHS was discontinued, the organization of farmers (The Federation of Swedish Farmers—LRF) continued by distributing folders about child safety on farms and other outreach activities. However, when there was a major national intervention program “Safe Farmers Common Sense” during 2009–2013, funded by the EU Rural Development program, the organizers learned that the best way to gain the attention of farmers was to start with the safety related to children. They produced a lot of educational materials and public messages, as well as books, games, and reflective jackets for children (6, 7).

### 2.2. Guidelines for children on farms

Inspired by the North American Guidelines for Children's Agricultural Tasks (NAGCAT) by Lee and Marlenga (8) a Swedish version was developed with a focus on children on animal farms (horses, pigs, and dairy cows) after a survey of the most common tasks performed by children on farms. The publication supplemented with checklists, and guidelines for first aid and other aspects were used by organizations of farmers and within agricultural schools (9).

A task not included in the Swedish guidelines is the use of farm tractors by children and a study by Pinzke et al. (10) regarding incidents on public roads concluded that the youngest tractor drivers aged 12–16 years were more often involved in road traffic incidents during school holidays and during harvest time. The over-representation of young children in tractor incidents suggests that it is questionable whether they should be allowed to operate farm vehicles without a driving license.

### 2.3. The farm parent's attitudes and perspectives on risk and exposure for their children

Research regarding the farm *parent attitudes to risk and injury to children* was done (11) and it was concluded that most parents know the risks on their farm, but are sometimes careless when working under stress or exhaustion. Some parents wanted more information and some wanted compulsory preventative or safety measures by manufacturers, e.g. a safety belt as standard on the extra seat in tractors. Children's friends were described as one of the greatest risks for injury due to peer pressure. Some parents mentioned that people who grow up on farms are sometimes “blind” to the dangers. Other parents seemed to overlook the risks and had their children carry out tasks for which they were not mentally or physically equipped. Some of the tasks the children reportedly carried out on farms conflicted with Swedish legislation.

Another study (4) had the focus on *parents' risk acceptance and attitudes toward the use of ATVs by children living in rural areas*. Major findings were that parents were aware of the risks and had a strong commitment to children's safety, but risk acceptance was a common issue, due to risk normalization. Parents did not see themselves as role models for children regarding the use of ATVs. To increase the safety of ATV use, recommendations to organizations and authorities were presented, such as an age limit for drivers of all adult-size ATVs and safety labeling of ATVs, with information clarifying the rules for specific vehicles.

It is also important to pay attention to *mental health and wellbeing among children living on farms*. In a recent study on rural crime and animal rights activism in Sweden by Ceccato et al. (12), it was concluded that also farm children are affected (worries, fear, and sleeping problems) by the increasing rural crime and by threats from animal rights activists toward animal farmers.

## 3. Current challenges

Living on a Swedish farm is definitely linked to a risky environment for children. A number of actions are needed in order to improve conditions. Farm parents need support and knowledge to safely raise their children. Small children need constant care and might need organized day care, which may not be available in rural areas. Innovative examples of farm families collaborating on childcare services might be a viable solution. In order to for farm parents to be more aware of risks and receive financial and educational resources, the general public should get involved. A promising, new national 5-year program will be launched in 2023 which intends to include the safety of children. A harder challenge is the pending closure of health and safety research within the Swedish University of Agricultural Sciences. In order to develop new knowledge and offer support to agricultural organizations there is a need for research that develops and tests childhood agricultural safety strategies, now and in the future.

## Author contributions

The author confirms being the sole contributor of this work and has approved it for publication.
